# Human Nitric Oxide Synthase—Its Functions, Polymorphisms, and Inhibitors in the Context of Inflammation, Diabetes and Cardiovascular Diseases

**DOI:** 10.3390/ijms22010056

**Published:** 2020-12-23

**Authors:** Magdalena Król, Marta Kepinska

**Affiliations:** Department of Biomedical and Environmental Analyses, Faculty of Pharmacy, Wroclaw Medical University, Borowska 211, 50-556 Wroclaw, Poland; m.krol1996@gmail.com

**Keywords:** nitric oxide synthase, oxidative stress, single nucleotide polymorphism, obesity, type 2 diabetes, heart diseases

## Abstract

In various diseases, there is an increased production of the free radicals needed to carry out certain physiological processes but their excessive amounts can cause oxidative stress and cell damage. Enzymes play a major role in the transformations associated with free radicals. One of them is nitric oxide synthase (NOS), which catalyzes the formation of nitric oxide (NO). This enzyme exists in three forms (NOS1, NOS2, NOS3), each encoded by a different gene. The following work presents the most important information on the NOS isoforms and their role in the human body, including NO synthesis in various tissues and cells, intercellular signaling and activities supporting the immune system and regulating blood vessel functions. The role of NOS in pathological conditions such as obesity, diabetes and heart disease is considered. Attention is also paid to the influence of the polymorphisms of these genes, encoding particular isoforms, on the development of these pathologies and the role of NOS inhibitors in the treatment of patients.

## 1. Introduction

During various processes taking place in the human body, free radicals and reactive oxygen species (ROS) are formed as natural metabolism products. Some of them act as signaling molecules that control physiological processes. Unfortunately, their excess can cause tissue damage [1]. For this reason, cells are forced to maintain a balance in the production of ROS to maintain homeostasis. To this end, compounds called antioxidants, which include a number of enzymes, including superoxide dismutase (SOD), catalase and glutathione peroxidase, play a role. However, maintaining control over these processes is not always possible. A condition in which too much ROS is produced and/or not effectively neutralized is referred to as oxidative stress. The imbalance between the excess of ROS and the biological ability to detoxify reactive products may accompany many pathological conditions, such as atherosclerosis or diabetes, but may also play a significant role in preventing aging as a result of mitohormesis [2].

In addition to ROS, reactive nitrogen species (RNS), a group of molecules derived from nitric oxide (NO), are characterized by high chemical reactivity due to having unpaired electrons [3]. They can, together with ROS, damage cellular structures. Their excessive production causes a phenomenon analogous to oxidative stress, referred to as nitrosative stress [4]. It is a state of imbalance between the amount of RNS formed and the biological capacity to render the reactive species harmless. It often damages proteins, lipids and even DNA, which can induce apoptosis. In turn, stronger nitrosative stress can cause necrosis. A high level of nitrosative stress reduces the adenosine triphosphate (ATP) pool, which prevents a cell from entering the path of controlled, apoptotic death, causing its necrosis [5].

NO is produced by a reaction catalyzed by nitric oxide synthase (NOS). Three isoforms of this enzyme, each associated with a different place of expression and action in the body, were distinguished [6]. Recently, there have been many reports on the impact of individual NOS isoforms and disturbances in their activity on the risk of various diseases, including metabolic and cardiovascular diseases. NO deficiency is one of the leading causes of endothelial dysfunction [7]. It is related to improper regulation of vasorelaxation, i.e., lowering the tone of blood vessels. In turn, such disorders are part of the pathogenesis of such diseases as atherosclerosis, hypotension, diabetes and hypercholesterolemia [8]. Attention can also be drawn to the importance of particular genotypes of selected polymorphisms of *NOS* genes. Nevertheless, there are still quite a few issues to ponder over or delve into more closely. This review was intended to inspire researchers to conduct further research, which can be translated into clinical significance. The aim of this study was to summarize the current knowledge of NO and NOS and their impact on selected disease states.

## 2. Characteristics of NO and Its Derivatives

The relevant compounds belonging to the RNS group are NO and its derivatives—nitrosyl cation (NO^+^), nitrosyl anion (NO^−^) and peroxynitrite (ONOO^−^). The most known NO derivatives are shown in Table 1. NO is formed from arginine in the reaction catalyzed by NOS. Although the half-life of NO is only a few seconds [9], it is a highly reactive molecule that, along with other free radicals, can cause the formation of new RNS, which, in turn, react with cell proteins and may impair their function due to the oxidation or nitrosylation of amino acid residues. NO acts as a cellular signaling molecule; it modulates muscle tone, regulates insulin secretion and modulates airway tone and intestinal peristalsis. In addition, it plays an essential role in angiogenesis and nerve development [10]. Dysregulated production of NO can lead to many pathological conditions, such as stroke, inflammation and hypertension. Therefore, NOS activity control using isoform-selective NOS inhibitors brings with it great hopes for treating diseases associated with NO production [11].

NO has long been known to be present in bacteria but for years there was no evidence for its biological functions in mammals [22]. Evidence since that time has established a significant role for NO as a messenger molecule in at least three systems: white blood cells, where NO mediates tumoricidal and bactericidal effects; blood vessels, where it represents endothelium-derived relaxing factor activity; and as a neuronal constituent with functions very much like those of a neurotransmitter [23].

In recent years, the study of the role of NO in cellular signaling has become one of the most rapidly growing biology areas. In many instances, NO mediates its biological effects by activating guanylyl cyclase and increasing cyclic guanosine monophosphate (cGMP) synthesis from guanosine triphosphate (GTP) [24]. However, the list of NO effects that are independent of cyclic GMP is also growing at a rapid rate; for example, NO can interact with transition metals such as iron, thiol groups, other free radicals, oxygen, superoxide anion, unsaturated fatty acids and other molecules [25]. Some of these reactions result in the oxidation of NO to nitrite and nitrate and terminate its effect, while different reactions can lead to altered protein structure, function or catalytic capacity. These diverse effects of NO, which are either cyclic GMP-dependent or independent, can change and regulate important physiological and biochemical events in cell regulation and function. NO can function as an intracellular messenger, autacoid, paracrine substance, neurotransmitter or as a hormone that can be carried to distant sites for effects [25]. Thus, it is a unique simple molecule with an array of signaling functions. However, as with any messenger molecule, there can be excess or deficiency of the substance resulting in pathological events.

In addition to NO, its derivatives are known to play a role in the pathophysiology of various diseases. It is worth paying attention here to the formation of RNS. Considered in terms of strict chemical criteria, RNS encompasses such a diverse range of compounds, with such contrasting and distinct properties, that their only unifying characteristic is that they can be derived from NO. Recent advances give essential insights into the biology of specific RNS, their effects on physiological functions and their potential participation in the development of diseases [26]. In biological systems, the primary source of all RNS is NO. The rapid reactions of NO with free radicals have proved to be significant routes to the formation of RNS and, at present, the best known of them is the reaction with peroxide (O^−2^) to produce ONOO^−^ [26,27,28]. Peroxynitrite is chemically unstable under physiological conditions resulting in the formation of nitrate through isomerization. Since nitrate is essentially biochemically inert in mammalian cells, this reaction has been shown to be an excellent method to scavenge and neutralize O^−2^ [29]. As studies on this reaction progressed, a new perspective emerged when researchers realized that ONOO^−^ is reactive with all the major classes of biomolecules and, therefore, has the potential to mediate cytotoxicity independently of NO or O^−2^ [30].

ONOO^−^ is a highly reactive species which can directly react with various biological targets and components of a cell, including lipids, thiols, amino acid residues, DNA bases and low-molecular-weight antioxidants [31]. However, these reactions happen at a relatively slow rate. This slow reaction rate allows it to react more selectively throughout the cell. Furthermore, ONOO^−^ can react with other molecules to form additional types of RNS, including nitrogen dioxide (•NO_2_) and dinitrogen trioxide (N_2_O_3_), as well as different types of chemically reactive free radicals [32]. ONOO^−^ can react with proteins that contain transition metal centers and, through this, modify proteins such as hemoglobin, myoglobin and cytochrome c. This molecule can change protein structure through reactions with various amino acids through cysteine oxidation or tyrosine nitration. However, ONOO^−^ does not react directly with tyrosine. Tyrosine reacts with other RNS produced by peroxynitrite. These reactions affect protein structure and function and can potentially cause changes in the catalytic activity of enzymes, alter cytoskeletal organization and impair cell signal transduction [33]. Such changes at the molecular level can underlie many diseases, such as cardiovascular diseases, diabetes, chronic inflammatory diseases and cancer, and neurodegenerative disorders.

## 3. The Structure of NOS and Its Isoforms

To fully understand the role and function of NO, it is necessary to become familiar with the enzyme responsible for its synthesis and the structural differences in the enzyme isoforms. NO synthases (NOS) are a group of enzymes that catalyze the synthesis of NO from the nitrogen residue of the amino acid L-arginine in the presence of nicotinamide adenine dinucleotide phosphate (NADPH) and molecular oxygen. NOS is an enzyme that binds to flavin adenine dinucleotide (FAD), flavin mononucleotide (FMN), heme, tetrahydrobiopterin (BH_4_) and calmodulin. To date, three different types of this enzyme have been found in mammals. We can distinguish two constitutive NOS isoforms, the activity of which depends on the Ca^2+^/calmodulin complex—NOS associated with signal transduction in central and peripheral neurons (NOS1, ncNOS, bNOS) and endothelial NOS (NOS3, eNOS, ecNOS) related to NO synthesis in blood vessels [34]. A separate gene encodes both isoforms. NOS1 is located on the longer arm of chromosome 12 at position 24.22 and encodes a protein of 1434 amino acids [35]. In turn, NOS3 is located on the longer arm of chromosome 7 at position 36.1 and encodes a protein of 1153 amino acids [36]. NOS1 is especially important for the brain and peripheral nervous system, where NO performs functions as a neurotransmitter, and has been implicated in neurotoxicity associated with stroke and neurodegenerative diseases, neural regulation of smooth muscle, including peristalsis, and penile erection [37]. In turn, NOS3 is responsible for the production of NO in the vascular endothelium [38], a monolayer of flat cells lining the interior surface of blood vessels [39]. NO produced by NOS3 in the vascular endothelium plays critical roles in regulating vascular tone, cellular proliferation, leukocyte adhesion and platelet aggregation [40]. Therefore, a functional NOS3 is essential for a healthy cardiovascular system. The third form of NOS was originally isolated and sequenced from mouse macrophages [41]; its activity depends on the Ca^2+^/calmodulin complex but does not require high Ca^2+^ levels. It is completely active at normal intracellular Ca^2+^ levels [34]. Inducible NOS (NOS2, iNOS, mNOS, macNOS) is involved in the immune response and synthesizes NO, which is an essential pro-inflammatory cytotoxic agent, as a defense mechanism; for example, NO is responsible for inhibiting the production of IL-12 and macrophages. Furthermore, its expression occurs due to various inflammatory cytokines, including IL-1, IL-2, TNFα and lipopolysaccharide (LPS). NOS2 can be produced in most nucleated cells of the body and plays a vital role in eliminating or suppressing intracellular pathogens, including viruses [42]. The gene encoding NOS2 is located on the longer arm of chromosome 17 at position 11.2 and encodes a protein of 1203 amino acids [43]. The dual role of NOS2 in cancer development is known in the literature. It depends on the local concentration of NOS2 in the tumor microenvironment or disease state. This NOS isoform modulates key issues such as malignant transformation, angiogenesis and metastasis. However, NO used by macrophages has a cytotoxic or cytostatic effect on cancer cells [44]. In fact, the role of NOS2 in the cancer process is very complex; hence we can talk about it as a tumor promoter and suppressor at the same time [44,45].

**Figure 1:**
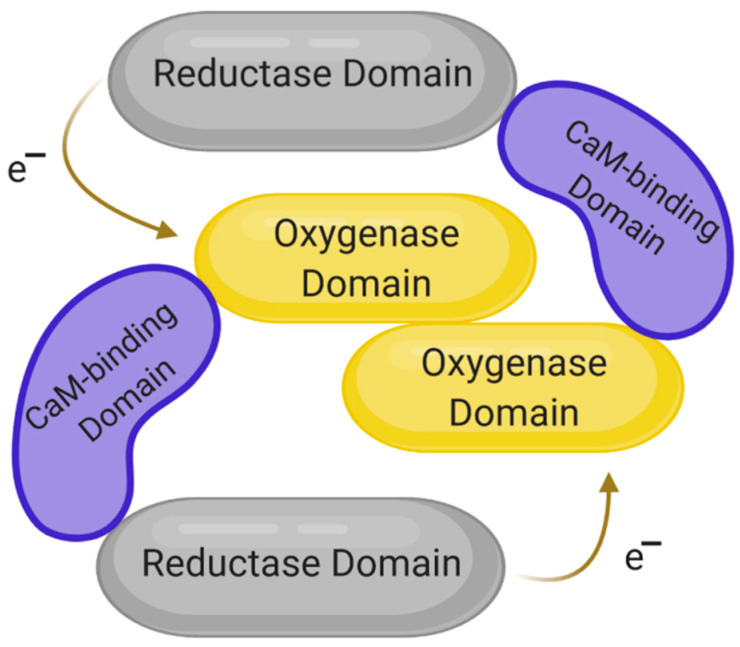
Schematic structure of nitric oxide synthase (NOS). Modified based on [49,50]. Created with BioRender.com. Legend: CaM—calmodulin; e^−^—free electron.

The structures of the three known isoforms are similar to each other; they are all dimers made of two identical subunits [46,47,48]. Each of the monomers has three domains: the reductase domain, the oxygenase domain and the calmodulin-binding domain, which is shown in Figure 1. The reductase domain consists of binding sites for FMN, FAD and NADPH, while the oxygenase domain is responsible for binding tetrahydrobiopterin (BH_4_). The task of the reductase domain, within which FMN and FAD play the role of functional groups, is to transport electrons from NADPH to the oxygenase domain of the opposite subunit. In turn, calmodulin binding is necessary to maintain the activity of each of the NOS isoforms [12,49].

All three isoforms catalyze the same reaction. In the first stage, the enzyme catalyzes the oxidation of L-arginine, thanks to which an intermediate compound—N-hydroxy-L-arginine—is formed, which is then oxidized to L-citrulline and NO is created [12,50].

**Figure 2:**
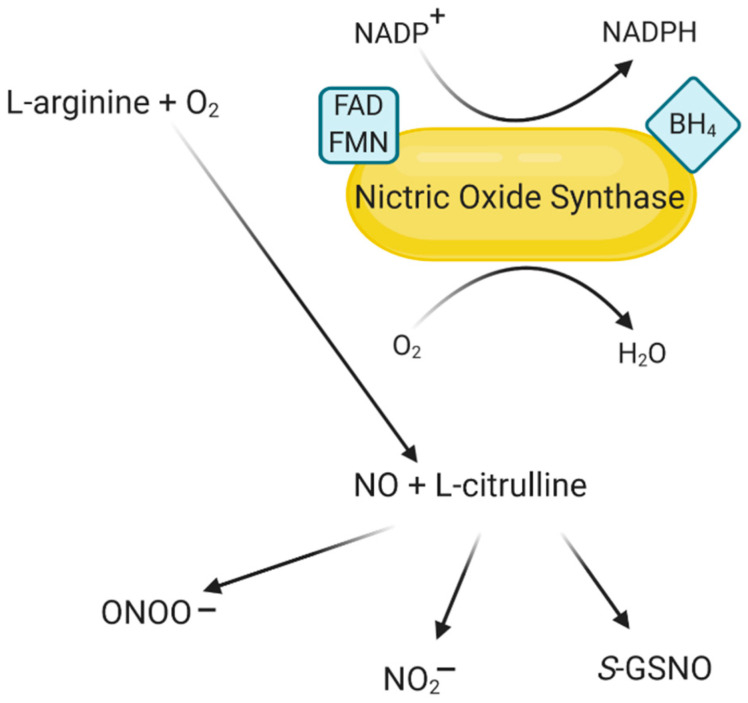
Synthesis of NO and related products. Modified based on [55,56,57]. Created with BioRender.com. Legend: FAD—flavin adenine dinucleotide; FMN—flavin mononucleotide; BH_4_—tetrahydrobiopterin; H_2_O—water; NADP^+^—glutamate dehydrogenase; NADPH—reduced nicotinamide adenine dinucleotide phosphate; O_2_—oxygen; S-GSNO—S-nitrosoglutathione.

NO, being a product of the NOS-catalyzed reaction, is also responsible for regulating this enzyme’s expression and activity. By reacting with the amino acid residues of the molecule and forming the S-nitroso group, NO can reversibly inhibit NOS activity [51]. Negative feedback of NO has been shown through a process called S-nitrosylation [52]. NOS1 and NOS2 can also undergo S-nitrosylation, although dynamic regulation of their function through such a route has not been proven. In addition, these two isoenzymes can form ferrous-nitrosylcomplexes in their heme groups, which causes their partial inactivation [53]. The factor limiting NO synthesis is the L-arginine substrate’s availability, which may be particularly important for cells in which the NOS2 isoenzyme is available [54]. NO synthesis catalyzed by NOS is shown in Figure 2.

## 4. The Role of NO and NOS in the Context of Inflammation, Diabetes and Cardiovascular Diseases

Although NO is a ubiquitous intercellular transmitter in all vertebrates, responsible for modulating blood flow and nervous activity, its excessive production can lead to nitrosative stress, leading to many pathological conditions. NO itself is not very toxic since the body is able to minimize the means that cause its accumulation. This action involves a group of scavenging enzymes, SOD or catalase, thanks to which NO is quickly removed by diffusion through tissues into erythrocytes. It is then transformed into nitrate by reaction with oxyhemoglobin [58,59].

NO derivatives, such as peroxynitrite (ONOO^−^), are much more potent oxidants. The ONOO^−^-forming reaction occurs very quickly and no enzyme is required. NO is the only known biological molecule that reacts very quickly with superoxide. At the same time, it is produced in such high concentrations that it can overcome endogenous levels of SOD and react with the superoxide before SOD removes it. It was formerly thought that NO alone directly attacks and damages cell DNA. It is currently believed that this effect depends precisely on the conversion of NO into higher nitrogen oxides. However, NO can reversibly inhibit transition metal enzymes or free radical intermediates in the catalytic cycle. It also demonstrates the ability to inhibit catalase and cytochrome P-450 reversibly. It can also inhibit ribonucleotide reductases, the enzymes responsible for DNA synthesis [50,58,59,60].

### 4.1. The Role of NO and NOS in Inflammation

In 1994, the relationship between the increase in NOS activity and inflammation development was shown [61]. The study involved NOS2 and cyclooxygenase (COX), which converts arachidonic acid into prostaglandin H2 that is then further metabolized to prostanoids. The parameters given were measured in the acute, chronic and receding stages of a mouse model of the granulomatosis air sac. COX and NOS2 activity were measured in acute phase skin samples for up to 24 h. Activities in granulomatous tissue were measured after 3, 5, 7, 14 and 21 days for chronic and resolving inflammation. The activity of tested NOS2 increased over 24 h on the skin and there was also a significant increase in granulomatous tissue between day 3 and day 7, followed by a decrease on day 14 and a further increase on day 21. However, in the chronic receding phase, decreased activity of both tested enzymes could be observed. This may indicate their diverse regulation, which may result from the changing cytokine pattern during the inflammatory response [61].

A few years later, other researchers also considered the role of NO and NOS in the immune response and inflammation [62]. It was already known that NO is synthesized by many cell types that are involved in immunity and inflammatory reactions. Ultimately, NO is an important molecule that participates in the body’s defense reactions against pathogenic microorganisms. The main enzyme involved in its production during inflammation is NOS2, which causes long-term NO synthesis at a high level. However, the role of NO in immune diseases and inflammation is still unclear. At high concentrations generated by NOS2, NO is rapidly oxidized to RNS, which mediate most of the effects of NO on the immune system. RNS can modify key signaling molecules, such as kinases and transcription factors, e.g., phosphoinositide 3-kinases (PI3K) [63]. They also inhibit several critical enzymes in mitochondrial respiration, for example nicotinamide adenine dinucleotide phosphate oxidase (NADPH oxidase) or monoamine oxidases (MAO), which leads to depletion of ATP and cellular energy [64,65].

The latest studies also focus on the role of NO in accompanying inflammation, including inflammatory joint disease and the role of this molecule in endothelial function. Endothelial dysfunction is attributed to a reduction in the biological activity of NO in rheumatoid arthritis (RA). However, the relationship between NO and endothelial inflammation and dysfunction in RA has not yet been thoroughly investigated and explained [66]. Research conducted by Garg et al. [67] showed that serum nitrite levels in RA patients were significantly higher compared to the control group. A positive correlation between the concentrations of nitrate, C-reactive protein (CRP) and TNF-α was also observed. These studies show that inflammatory disease activity and endothelial dysfunction in RA are associated with increased levels of pro-inflammatory cytokines and NO. The release of cytokines induced the production of NO, which mediates endothelial dysfunction. Therefore, it should be noted that NO plays an essential role in inflammation-induced endothelial dysfunction in RA [67].

### 4.2. The Role of NOS in Obesity

At the beginning of the 21st century, it was shown that NO is produced in adipose tissue and that lipolysis can be inhibited by this molecule. One of the studies included obese men who had NOS expression analyzed in the subcutaneous fat [68]. The results showed that NOS2 and NOS3 mRNA expression was detected in isolated fat cells and pieces of adipose tissue. The mRNA of NOS1, however, was not detected. Hormone-sensitive lipase (HSL), the enzyme responsible for regulating lipolysis, showed reduced activity in obese people. The expression of HSL in the subcutaneous fat was also examined in the same subgroup of patients. According to the results of this study, HSL levels were reduced in obese patients. The study showed that NOS2 and NOS3, but not NOS1, were present in human subcutaneous fat. In addition, NOS3 expression and NOS3 protein levels were increased in obese patients, while HSL protein levels were reduced. Increased NO production and reduced HSL levels may be able to induce reduced lipolysis of subcutaneous fat in obesity [68].

A few years later, intensive work began on the inhibition of NOS2 that would contribute to the treatment of obesity. The study involved obese mice that had reduced scatter sensitivity to satiety signals [69]. The nodose ganglia and jejunum were analyzed by immunoblotting for NOS2 expression. NOS2 expression and NO production were found to be increased in the nodose ganglia and the small intestine in obese mice. It was also observed that NOS2 pretreatment with inhibitors—L-NIL (hydrochloride) and *N*^ω^-propyl-L-arginine—in obese mice increased the excitability of the nodal neuron and thus restored afferent sensitivity to satiety signals and reduced short-term energy intake. In obese mice given NOS2 inhibitors daily for three weeks, reduced energy intake and reduced weight gain in the first week and less epididymal fat at the end of three weeks were seen compared to saline mice. The results of these studies show that inhibiting NOS2 or blocking the action of NO on afferent pathways can be used to treat obesity.

In 2020, Udi et al. [70] investigated the effect of a new hybrid inhibitor—dual cannabinoid receptor type 1 (CB_1_ receptor)/NOS2 antagonist—on the relief of obesity-induced chronic kidney disease (CKD). To this end, said formulation was orally administered to mice for 28 days. The inhibitor was shown to reduce morphological and functional changes in the kidneys caused by obesity by reducing kidney inflammation, fibrosis, oxidative stress and kidney damage. This study shows that blocking CB_1_ receptors and NOS2 may be of great therapeutic importance in alleviating obesity-related CKD.

Research on the relationship between gene polymorphisms encoding NOS isoforms and the development of obesity is inconsistent. However, the polymorphism of the NOS3 (rs2070744) seems to be of significant interest [71]. The influence of this polymorphism on metabolic syndrome (MetS) in obese children and adolescents has been demonstrated. The distribution of NOS3 genotypes in the studied groups was compared. It has also been shown that the CC genotype of the rs2070744 polymorphism is associated with MetS in obese children and adolescents [72]. However, more research is certainly needed on the influence of NOS3 polymorphisms and other genetic markers on the risk of developing metabolic diseases.

Finally, Teixeira et al. [73] investigated the kinetic response of NO after a session of acute eccentric resistance exercise (ERE) and the possible effect of the rs1799983 polymorphism in the NOS3 in elderly obese women. To this end, 87 women completed seven sets of ten eccentric repetitions at 110% of the ten maximum repetitions. The group with the GG genotype was characterized by higher body weight, obesity, higher BMI and relatively higher muscle strength, with significantly lower concentrations of triglycerides, VLDL and urea compared to the groups with the TT and TG genotypes. Hence, carriers of T should pay more attention to cardiovascular risk factors and metabolic disorders.

### 4.3. The Role of NOS in Insulin Resistance and Diabetes

Expression of all three NOS isoforms can be detected in pancreatic β cells. NOS1 is located mainly in the secretory granules of insulin and in the mitochondria and the cell nucleus [74]. In contrast, NOS2 is not detectable in β-cells at basal glucose levels; its expression occurs only after exposure to higher glucose concentrations [75,76]. However, it is known that in the states of insulin resistance, NOS2 participates in the deregulation of metabolic processes of tissues by disturbing the balance in glucose and lipid homeostasis and endothelial dysfunction through the creation of local and systemic inflammatory environments [77]. This is due to increased nitrosation stress, which affects the action of proteins involved in the maintenance of metabolism and vascular homeostasis through cysteine S-glutathionylation and the nitration of tyrosine residues of other vital proteins. The presence of NOS3 in pancreatic cells has also been confirmed but there is still too little data on its function [78].

An important issue is also the role of mitochondria in NO generation. It is well known that NO can act as an inducer of mitochondrial permeability transition (MPT) through its direct effect on MPT pores [79]. Additionally, NO may induce indirect effects secondary to the inhibition of oxidative phosphorylation, which may trigger apoptosis by inducing mitochondrial membrane permeabilization [80]. These activities may contribute to the development of diabetes mellitus, as diabetes is closely associated with changes in the structure and function of the mitochondria at the cellular level [81,82]. Disruption of glucose uptake, which is the primary source of energy, disrupts the metabolism of cellular energy and thus the functioning of the mitochondria. Thus, the mutual connection between the functioning of mitochondria and the pathophysiology of diabetes is visible. Moreover, the development of oxidative stress accompanies both diabetes and the induction of MPT pores. On this basis, it can be concluded that the MPT pores are directly involved in the pathology of diabetes [83]. However, additional studies are needed to clearly confirm or rule out this thesis.

NO from NOS1 and NOS3 can act as a mediator or inhibitor in the negative feedback associated with glucose-stimulated insulin secretion (GSIS) [84]. In addition, NOS1-derived NO increases glucokinase (GK) activity through S-nitrosylation of cysteine residues, a critical process that mediates the dissociation of GK from and enhances insulin secretion [85]. In isolated islets of Langerhans, increasing the glucose dose increases the activity of all three NOS isoforms. However, the activity of NOS1 and NOS3 is more quickly adapted to increasing glucose concentration [86]. Inhibition of the activity of these two isoforms in islets of Langerhans enhances GSIS. This negative feedback effect inhibits excessive insulin secretion in response to high glucose levels and protects pancreatic β cells. One possible mechanism of NO negative feedback on GSIS is the inhibition of phospho-fructokinase and glucose metabolism in pancreatic β-cells [87]. Apart from the production of NO, NOS1 also exhibits cytochrome C reductase activity [88]. NOS1 inhibits GSIS by increasing NO production and stimulating GSIS through its nonoxidative activity; a balance between these two activities is essential for proper insulin secretion in response to glucose [89].

NOS2 protein expression is high in pancreatic islets of patients with type 2 diabetes (T2D) and inhibition of NOS2 expression restores disturbed GSIS [90]. NO derived from NOS2 most often causes β-cell dysfunction, impaired insulin secretion, hyperglycemia and the development of diabetes [91]. NOS2, through the cGMP-independent mechanism, inhibits insulin secretion [87] as a result of inhibition of the mitochondrial electron transport chain (complexes I and II) and the activity of mitochondrial aconitase [92], S-nitrosylation of critical thiol groups involved in the secretory process [93] and also tyrosine nitration and subsequent GK regulation [94].

The hypothalamic NOS–NO system is involved in the central regulation of glucose homeostasis and NOS1 is the main isoform involved in this process [95]. The central NOS–NO system regulates insulin secretion and its peripheral action and the acute blockage of NOS in the CNS causes hyperglycemia, peripheral insulin resistance and decreased insulin secretion [96]. Elevated NO concentrations in the hypothalamus lead to liver insulin resistance and increased GSIS [97]. However, the mechanisms by which the central NOS–NO system regulates insulin secretion and mediates the effects of insulin on peripheral tissues are not yet well understood.

Endothelial dysfunction, such as impaired NO production, is considered an early stage in the development of insulin resistance, atherosclerosis and T2D [98]. Studies assessing the relationship between ischemic heart disease (CHD) and endothelial dysfunction have clearly shown that reduced NO-dependent endothelial vasodilatation is an early functional disorder in the development of atherosclerotic lesions [99]. Moreover, as previously mentioned, among the many effects of NO, its ability to modulate peripheral and hepatic glucose metabolism and insulin secretion has also been demonstrated. It has also been suggested that changes in NO play an important role in the development of insulin resistance and T2D [100].

In T2D, polymorphisms in the NOS3 seem to attract particular attention, such as the tandem repeat polymorphism (VNTR) of the NOS3, which has been associated with the development of diabetic nephropathy. A study involving Japanese patients showed that the VNTR of the NOS3 might be associated with the progression of diabetic nephropathy in people diagnosed with T2D [101].

Another study focused on assessing *NOS3* polymorphisms in the context of the risk of diabetic nephropathy concerned the analysis of polymorphisms: rs1799983, rs2070744 and 27-bp VNTR [102]. A total of 400 patients with T2D were enrolled in this study. The group with diabetic nephropathy consisted of 200 patients; the group with diabetes without nephropathy also consisted of 200 patients. Genetic analysis of the *NOS3* polymorphisms was carried out for all subjects. The T allele of the rs1799983 polymorphism and the C allele of the rs2070744 polymorphism were significantly more frequent in diabetic nephropathy patients than in patients without nephropathy. However, in the case of the 27-bp VNTR polymorphism, there was no significant change in NO concentrations in the different genotyping groups of patients with diabetic nephropathy and without it. The obtained results suggest that the *NOS3* polymorphisms could indeed constitute genetic determinants of the development of diabetic nephropathy in patients with T2D in the studied population

### 4.4. The Role of NOS in Heart Disease

NOS1 is the primary endogenous source of NO for the heart, facilitating myocardial relaxation and modulating contractions. Therefore, this isoform plays a key role in protecting the myocardium against the effects of increased oxidative stress, systolic/diastolic dysfunction, adverse structural remodeling and heart failure disorders. In a healthy heart, NOS1-derived NO attenuates the underlying inotropy of the heart by modulating L-type calcium channel (LTCC) activity in the plasma membrane to reduce the amplitude of intracellular Ca^2+^ transition states [103] through S-nitrosylation or cGMP-dependent mechanisms. In the sarcoplasmic reticulum, NO facilitates myocyte relaxation by promoting intracellular Ca^2+^ reuptake [104]. NO derived from NOS1 can be activated directly by S-nitrosylation [105] or indirectly by peroxynitrite-dependent S-glutathionylation [106]. In addition, NOS1 may affect myocardial function by regulating mitochondrial proteins. It has been reported that NO derived from NOS1 inhibits the mitochondrial respiratory chain, including complexes I, III and IV [107,108,109], and reduces mitochondrial oxygen consumption, thus affecting heart metabolism.

In states like ischemia-reperfusion injury [110], infarction [111], hypertrophy and heart failure [112,113], the expression and activity of NOS1 are increased. Various studies indicate that NO from NOS1 prevents diastolic dysfunction, increases the β-adrenergic reserve, reduces left ventricular hypertrophy and protects the heart against arrhythmogenesis [114]. Zhang et al. [114] suggest that the increase in NOS1 concentration and activity is an early event after pathogenic trauma and during disease progression and that NOS1 has a protective function in the heart. It has been shown that acute in vitro treatment with angiotensin II of isolated left ventricular (LV) myocytes significantly increases the expression and activity of NOS1. In turn, NOS1-derived NO decreased NADPH oxidase superoxide production and facilitated the relaxation of LV myocytes through cGMP/PKG-dependent PLN-Ser16 phosphorylation. Similarly, NOS1 expression and activity were increased in the LV myocytes of rats with Ang II-induced early hypertension. NOS1 activity was equal to the ratio of phosphorylated NOS1 levels to total NOS1 levels [115].

Nishijima et al. [116] investigated the effect of NOS2 on atrial oxidative stress and electrophysiological changes in the heart in dogs. To this end, the animals were divided into two groups—one received a placebo while the other received active treatment (NOS cofactor, BH_4_ and NOS substrate). Heart failure increased atrial NOS2 and decreased atrial BH_4_, while L-arginine remained unchanged. Heart failure resulted in left atrial oxidative stress that was weakened by treatment with BH_4_ and L-arginine. This indicates that chronic ischemic heart failure leads to atrial oxidative stress and electrophysiological abnormalities through BH_4_ depletion and NOS2 decoupling. Thus, modulation of NOS2 activity by supplementing BH_4_ may be an effective way to reduce the frequency of atrial arrhythmias.

Research confirms that the other two NOS isoforms may also be associated with the pathophysiology of heart disease. Liu et al. [117] evaluated the role of NOS3 in the pathogenesis of hypoplastic coronary arteries. For this purpose, they used three groups of mice—wild-type (WT), NOS3-deficient and mice with heart-specific NOS3 overexpression. NOS3 deficiency resulted in coronary artery hypoplasia in fetal mice and spontaneous myocardial infarction in postpartum hearts. In NOS3-deficient mice at birth, significant reductions in coronary artery diameter, vessel density and volume were found. In addition, NOS3-deficient mice showed substantial increases in ventricular wall thickness, myocardial volume and cardiomyocyte size compared to WT mice. Therefore, it should be assumed that NOS3 is essential for the development of coronary arteries and that its deficiency leads to hypoplastic coronary arteries.

It is known that single nucleotide polymorphisms (SNPs) in NOS are associated with the cardiovascular system’s pathophysiology. Levinsson et al. [118] investigated NOS variants’ association with CHD and hypertension. For this purpose, 560 people diagnosed with CHD were genotyped at 58 selected SNPs in the NOS that were most strongly associated with the aforementioned ailments. It turned out that the SNP of NOS1, rs3782218, showed the most consistent association with both phenotypes. In turn, the association with CHD was observed with two other SNPs—those of NOS1 (rs2682826) and NOS3 (rs1549758). In the case of arterial hypertension, additional SNPs were observed, including the SNP of NOS3, rs3918226. It was thereby confirmed that NOS1 is the most important risk gene for NOS-dependent coronary artery disease.

However, another study showed an association between NOS polymorphisms and increased susceptibility to the development of atherosclerosis and coronary artery disease (CAD). For this purpose, the researchers tested for the 27-bp tandem repeat polymorphism (VNTR) in intron 4 of the *NOS3* in 141 unrelated CAD patients with positive coronary angiograms and 159 age-matched controls with no symptomatic history of CAD [119]. Although the frequency of different genotypes of this polymorphism differed significantly between patients with CAD and the control group, it cannot be determined whether this polymorphism was an independent factor in developing the studied disease. For this purpose, more detailed studies should be conducted on a larger number of subjects.

Selected polymorphisms of the various NOS isoforms, along with their associated diseases, are presented in Table 2.

## 5. Selected NOS Inhibitors and Their Role in Therapy

As already explained, NO plays a vital role in the homeostasis of various physiological systems, including micro- and macrovascularization, inhibition of platelet aggregation and regulation of neurotransmission in the central nervous system and gastrointestinal, respiratory and genitourinary systems. However, its overproduction is associated with many diseases, such as arthritis, asthma, cerebral ischemia, Parkinson’s disease, neurodegeneration and seizures [125]. For this reason, greater interest should be directed to the design of NOS inhibitors with therapeutic purposes.

The first designed NOS inhibitors appeared in the 1980s and 1990s and were based on L-arginine, an enzyme substrate. This approach led to strong compounds but, unfortunately, with a poor level of selectivity among the isoforms. At the end of the 1990s, the first crystal structures of NOS2 and NOS3 were revealed, showing a high degree of similarity, especially in their active site. One of the most critical moments in the history of NOS inhibitors was the description of highly selective NOS2 inhibitors by Garvey et al. in 1994 [126]. The compounds were isothiourea derivatives designed as reversible inhibitors that competed with the L-arginine of human NOS2, with 190-fold selectivity compared to NOS3 but only about 5-fold compared to NOS1 [126,127]. However, further studies of this group led to the design of a highly selective compound for both NOS2 and NOS3 that could penetrate cells and tissues [127,128]. The crystal structure of NOS1 was described in 2002 and this achievement made it possible to design selective inhibitors [129,130]. NOS isoforms were approved as targets for new drugs shortly after their X-ray crystallography became available. Since then, the design of effective and selective inhibitors has become an important approach in developing modern drugs that cover the biochemical pathways of NO associated with many dysfunctions of the human body [131,132,133].

A number of NOS inhibitors have already been evaluated in clinical trials. Some of them are presented in Table 3. One of them was so-called tilarginine, a nonselective L-NMMA compound that has been assessed in North America and Europe. The administration of a 1 mg/kg bolus and a 5-h infusion did not reduce mortality in patients with refractory cardiogenic shock complicating myocardial infarction despite an open infarction artery. Although good results were shown in phase II, it was not successful in phase III [134,135]. In another study, L-NMMA showed no differences in mean arterial pressure (MAP) after 2 h compared to the placebo group [136].

However, when evaluating another inhibitor, N(G)-nitro-L-arginine methyl ester, in the treatment of refractory cardiogenic shock, it was shown that death after one month was 27% in the study group compared to 67% in the control group [137]. Further studies have been conducted to verify this; however, it was found that nonselective NOS inhibitors were of no clinical interest [147].

It should certainly be emphasized that the research on NOS inhibitors clearly moved forward, all thanks to X-ray crystallographic studies of this enzyme. This helps, in structure-based design approaches, in the search for selective inhibitors and in understand their mechanisms of action. Regardless, efforts have been made to give them a drug-like profile.

## 6. Conclusions

It is well recognized that NO has an essential role in many physiological processes. However, disturbances in their production, caused, e.g., by altered concentration or activity of NOSs, may drive the development of many pathologies. The relationship between the occurrence of specific polymorphisms of genes encoding isoforms of NOS and the development of insulin resistance has been demonstrated. Several genetic variants in the *NOS3* locus are associated with the development of type 2 diabetes and susceptibility to other metabolic complications. In turn, polymorphisms in *NOS2* are associated with a higher plasma glucose concentration and variants in the promoter sequence of this gene also correlate with type 2 diabetes. However, polymorphisms of the genes encoding NOSs are not only associated with metabolic disorders. Certain changes in *NOS1* appear to be related to high blood pressure or heart diseases such as CHD.

As can be seen from the examples above, the genetic changes associated with NOS can affect several organs or systems. Therefore, research on the polymorphisms of genes encoding NOS isoforms seems interesting and justified. Such studies may contribute to a better understanding of the molecular disorders that occur in specific disease entities. In addition, although many of these diseases are very common and many studies are carried out on them, many processes in the human body have still not been fully explained yet. Focusing on these topics can help confirm the role of genetic predisposition in the populations with these conditions. Furthermore, studies on the effects of NOS inhibitors in treating various diseases also seem to be necessary; however, to confirm their effectiveness, additional or more extended clinical trials are required.

## Figures and Tables

**Table 1:** The most well-known nitric oxide (NO) derivatives [12,13,14,15,16,17,18,19,20,21].

Molecule Name	Summary Formula	Reactivity	Characteristics
nitrosyl cation	NO^+^	a strong oxidizing agent	Intermediate in the amine diazotization reaction
nitrosyl anion	NO^−^	a strong oxidizing agent	Participates in the nitrosylation reaction of metals, forming metal nitrosyl complexes
nitrogen dioxide	•NO_2_	a strong oxidizing agent	A good oxidizer; it combusts, sometimes explosively, with many compounds, such as hydrocarbons
dinitrogen trioxide	N_2_O_3_	a strong oxidizing agent	Partially dissociates into NO and NO_2_; vapors very toxic by inhalation; reactivity likely to resemble that of nitrogen dioxide
peroxynitrite	ONOO^−^	highly reactive; a very strong oxidant and nitrating agent	Essentially stable but its protonated form (ONOOH) decomposes rapidly via homolysis of the O-O bond to form about 28% free NO_2_ and OH radicals
nitrite	NO_2_^−^	very reactive; a member of reactive nitrogen species	A nitrogen oxoanion formed by loss of a proton from nitrous acid; used for NO measurement
nitrate	NO_3_^-^	very reactive; a member of reactive nitrogen species	A nitrogen oxoanion formed by loss of a proton from nitric acid; used for NO measurement
nitroxyl	HNO	very reactive towards nucleophiles, including thiols	A weak acid; can be formed as a short-lived intermediate in the solution phase

**Table 2:** Selected polymorphisms of NOS and their characteristics [71,72,73,101,102,118,119,120,121,122,123,124].

Polymorphism	Isoform	Location	Disease	References
rs3782218	NOS1	C2637T	ischemic heart disease, hypertension	[118]
rs2682826	NOS1	C276T	ischemic heart disease	[118]
rs2779248	NOS2	T278C	type 2 diabetes	[120,121]
rs1137933	NOS2	C231T	type 2 diabetes	[120,121]
Tandem repeat polymorphism (VNTR)	NOS3	27-bp VNTR	diabetic nephropathy, acute eccentric resistance exercise, metabolic syndrome, atherosclerosis, coronary artery disease	[101,102,119]
rs1799983	NOS3	G894T	diabetic nephropathy	[73,102,122]
rs2070744	NOS3	T786C	diabetic nephropathy	[71,72,102]
rs1549758	NOS3	C774T	ischemic heart disease, coronary artery disease	[118,123]
rs3918226	NOS3	C665T	hypertension	[118,124]

**Table 3:** NOS inhibitors and research conducted on them [134,135,136,137,138,139,140,141,142,143,144,145,146].

Inhibitor Name	Country/Continent	Application in Clinical Test/Research	References
Tilarginine (L-NMMA)	North America, Europe	Patients with cardiogenic shock; patients with breast cancer	[134,135,136]
N(G)-nitro-L-arginine methyl ester (L-NAME)	North America, Asia	Patients with cardiogenic shock; patients with septic shock	[137,138]
Asymmetric dimethylarginine (ADMA)	Europe	Possible use as a cardiovascular risk factor	[139,140]
N(G)-methyl-l-arginine hydrochloride	Europe	Used to restore the balance of vasomotor tone in patients with septic shock	[141,142]
7-nitroindazole (7-NI)	Europe, North America	Anticonvulsive properties in seizure models in rodents	[143,144]
Aminoguanidine	Asia	Alleviation of graft-versus-host disease in mice; alleviation of the susceptibility of mice to bacterial infections	[145,146]

## Data Availability

Data sharing not applicable. No new data were created or analyzed in this study. Data sharing is not applicable to this article.
